# A Multimodal Ensemble Driven by Multiobjective Optimisation to Predict Overall Survival in Non-Small-Cell Lung Cancer

**DOI:** 10.3390/jimaging8110298

**Published:** 2022-11-02

**Authors:** Camillo Maria Caruso, Valerio Guarrasi, Ermanno Cordelli, Rosa Sicilia, Silvia Gentile, Laura Messina, Michele Fiore, Claudia Piccolo, Bruno Beomonte Zobel, Giulio Iannello, Sara Ramella, Paolo Soda

**Affiliations:** 1 Research Unit of Computer Systems and Bioinformatics, Department of Engineering, Università Campus Bio-Medico di Roma, Via Àlvaro del Portillo, 21, 00128 Roma, Italy; 2 Department of Computer, Control, and Management Engineering, Sapienza University of Rome, 00185 Roma, Italy; 3 Operative Research Unit of Radiation Oncology, Fondazione Policlinico Universitario Campus Bio-Medico, Via Alvaro del Portillo, 200, 00128 Roma, Italy; 4 Operative Research Unit of Diagnostic Imaging, Fondazione Policlinico Universitario Campus Bio-Medico, Via Alvaro del Portillo, 200, 00128 Roma, Italy; 5 Research Unit of Radiation Oncology, Department of Medicine and Surgery, Università Campus Bio-Medico di Roma, Via Àlvaro del Portillo, 21, 00128 Roma, Italy; 6 Research Unit of Diagnostic Imaging, Department of Medicine and Surgery, Università Campus Bio-Medico di Roma, Via Àlvaro del Portillo, 21, 00128 Roma, Italy; 7 Department of Radiation Sciences, Radiation Physics, Biomedical Engineering, Umeå University, 901 87 Umeå, Sweden

**Keywords:** multimodal deep learning, multiexpert systems, optimisation, convolutional neural networks, precision medicine, oncology, medical imaging, tabular data

## Abstract

Lung cancer accounts for more deaths worldwide than any other cancer disease. In order to provide patients with the most effective treatment for these aggressive tumours, multimodal learning is emerging as a new and promising field of research that aims to extract complementary information from the data of different modalities for prognostic and predictive purposes. This knowledge could be used to optimise current treatments and maximise their effectiveness. To predict overall survival, in this work, we investigate the use of multimodal learning on the CLARO dataset, which includes CT images and clinical data collected from a cohort of non-small-cell lung cancer patients. Our method allows the identification of the optimal set of classifiers to be included in the ensemble in a late fusion approach. Specifically, after training unimodal models on each modality, it selects the best ensemble by solving a multiobjective optimisation problem that maximises both the recognition performance and the diversity of the predictions. In the ensemble, the labels of each sample are assigned using the majority voting rule. As further validation, we show that the proposed ensemble outperforms the models learning a single modality, obtaining state-of-the-art results on the task at hand.

## 1. Introduction

Lung cancer is the second most common type of tumour worldwide, accounting for approximately 11.4% of all cases [1], and it is the first in terms of number of deaths. Non-small-cell lung cancer (NSCLC) is the most frequent, with approximately 82% of all cases [2]. The most common treatment options, selected according to patients’ characteristics, include radiotherapy, chemotherapy, surgical resection, and immunotherapy but also targeted therapy [2,3].

Overall survival (OS), a measure of the time elapsed from the date of diagnosis until the patient’s death, allows the identification of subgroups of patients with a better or worse prognosis. Nevertheless, the 5-year survival rate for NSCLC is 26%, and it drops further to 7% when local recurrence or distant metastases occur [2]; in this respect, strategies to improve OS are urgently needed.

Over the last few years, there has been a growing interest in the development and application of Artificial Intelligence (AI) methods to oncology to help personalised medicine make further progress by facilitating the identification of the correct treatment for each patient. This has fostered the emergence of radiomics, which represents the bridge between medical imaging and personalised medicine since it computes, in a non-invasive manner, quantitative characteristics from medical images, such as CT, MRI, X-ray, and PET, representing tumour phenotype [4,5,6,7]. In addition to radiomics, researchers have attempted to extract prognostic information from other modalities, e.g., genome sequencing, whole-slide images (WSI), etc. [8,9,10]. For example, genomics data from a tumour allow the identification of cancer driver genes, whilst a WSI from a biopsy provides insight into the morphology and microenvironment of the tumour.

Several learning methods exist to perform these prognostic tasks, which can be roughly divided into model-based and data-based approaches. The former assume a model to describe the data trend, whilst the latter, exploiting the current large availability of digital repositories and using increasingly high-performance AI algorithms, learn directly from the data. In lung cancer predictive applications, such learning methods usually exploit one modality only [11,12,13,14,15,16], but the availability of multimodal data, which provide complementary information about the phenomenon under investigation, has led to the development of multimodal learning techniques able to cope with different information and to perform significantly better than unimodal models [17,18,19,20,21]. From an AI perspective, early, joint, and late fusion are the three main fusion techniques to merge different modalities’ information. In the first technique, the features of each modality are merged according to a rule into a feature vector to be given to the learner; in the second, the different modalities are merged at hidden and embedded levels, whilst in the last technique, the predictions made using the individual modalities are aggregated according to an aggregation rule.

In NSCLC, several studies have searched for a set of quantitative biomarkers, also referred to as a signature, to predict the overall survival. Among them, Table 1 summarises those using multimodal approaches [22,23,24,25], which are also now shortly overviewed.

In Amini et al. [22], the authors used the NSCLC dataset available on The Cancer Imaging Archive (TCIA) [26] to present an early fusion-like approach which fuses PET and CT images, using a technique based on 3D discrete wavelet transform to combine spatial and frequency features, and then it extracts radiomic features (first-order, textural, and moment invariant features). After performing feature selection via univariate Cox analysis, the authors applied the Kaplan–Meier method. The proposed approach obtained a concordance index (C-index) of 0.708, measured with 1000-time bootstraps, which is higher than the results they achieved from unimodal and traditional early fusion approaches (concatenation and averaging of the feature vectors separately extracted for each modality).

In Wu et al. [23], the authors used another NSCLC dataset also available on TCIA [27], and they performed an early fusion of deep features extracted from CT images and clinical data. The former were extracted using a 3D-ResNet34, whilst the latter using a Multilayer Perceptron (MLP). The concatenation of these features fed an MLP. In 5-fold cross-validation with a patient-level split, the authors tested different configurations by varying the structure of the ResNet, the depth of the final MLP, and the ratio between the number of the two types of deep features, achieving a C-index equal to 0.658 as best result.

In He et al. [24], the authors developed a hierarchical multicriterion fusion strategy to combine the predictions made by various classifiers working with different modalities. Even this study is based on the same data available on TCIA [27] used by [23], and it only takes into account 316 patients in whom the gross tumour volume was delineated. This permitted to extract clinical features and radiomic features (textural and non-textural) for each patient that, after a feature selection step separately performed for the two modalities were fed into the system. The modular architecture allows each modality to be analysed separately with a set of classifiers (Support Vector Machine, k-Nearest Neighbours, Decision Tree, Random Forest, and Extreme Gradient Boosting). By means of a sequence of aggregation rules that weight the contribution of each classifier to the output probability of each modality and then combine the probabilities of each modality, the system produces the final prediction. The experiments, run in 5-fold cross-validation, return an Area Under the ROC Curve (AUC) equal to 0.81.

In Vale-Silva and Rohr [25], the authors used the data in the National Cancer Institute’s Genomic Data Commons database [28] to develop a multimodal deep learning method for long-term pan-cancer survival prediction, called MultiSurv, which works with six different modalities, namely clinical data, gene expression, microRNA expression, DNA methylation, gene copy number variation data, and WSI. In this modular architecture, each input data modality is handled by a dedicated submodel. For the clinical and omics submodels, they used an MLP, whilst for the imaging submodel a ResNeXt-50. The data fusion layer aggregates the multimodal feature representations by taking the element-wise maxima across the set of representation vectors, allowing any missing modalities to be handled as well. The fusion vector is the input to an MLP, which returns as output a vector of probabilities, one for each time interval of a set of predefined follow-up time intervals. This system was trained in an end-to-end fashion, applying an holdout cross-validation stratified by cancer type. The authors evaluated the model with different numbers and combinations of the six modalities, and the best performance was obtained with bimodal inputs combining clinical data with gene expression (time-dependent C-index: 0.822).

Although the works in the literature achieved promising results, they are few in number, despite the importance of predicting the overall survival in NSCLC cancer that, in turn, may open the chance to develop personalised therapeutic approaches. Furthermore, two out four of such contributions explored early fusion, one investigated late fusion, and the other joint fusion. In particular, the one using late fusion computes handcrafted features from CT images that feed well-established classifiers. Nevertheless, in the last decade, deep learning has shown its potential in several fields, medical imaging included [29,30,31], to automatically learn discriminative features directly from images, without being limited to using predefined features or other descriptors whose definition come from researchers’ experience. In particular, Convolutional Neural Networks (CNNs) are a well-established set of network architectures exploiting convolutional layers (and their variations) to learn a compact hierarchical representation of the input that well fits the specific task to solve. In this respect, and as an evolution of the state-of-the-art shown in Table 1, in this work, we present a method to algorithmically optimise the way to set up a multimodal ensemble of deep networks, which are then combined by a late fusion approach. Such an ensemble uses image and clinical data to tackle the challenge to predict the overall survival in a cohort of 191 patients affected by NSCLC cancer. Exploiting the classifications of different unimodal models, we propose an optimised multimodal late fusion approach, whose performance is shown in Section 4. In particular, our method addresses a key and open question in multimodal deep learning [18,32], i.e., which should be the deep networks for each modality to be combined in the ensemble among the many available.

The manuscript is organised as follows: the next section describes the materials, and Section 3 introduces the methods. Section 4 presents and discusses the experimental results; finally, Section 5 provides concluding remarks.

## 2. Materials

Our clinical decision support system uses image and clinical data available within the CLARO dataset, which includes 191 NSCLC patients treated with concurrent chemoradiation for locally advanced NSCLC (86% of cases) and systemic treatment in the metastatic setting (14%). During treatment, all patients underwent weekly chest Computed Tomography (CT) scans, without intravenous contrast, to assess acute toxicity and tumour shrinkage, which were reviewed by two radiation oncologists independently. For all CTs, each physician was able to judge whether reduction was: (a) present and clinically significant, (b) present and clinically non-significant, or (c) absent. In the case of physician agreement for the (a) category, a contrast-enhanced CT was performed to better visualise node reduction, a new target volume was delineated, and a new treatment plan performed. Patients were treated without any time break.

The population was enrolled under two different approvals (the retrospesctive and prospective phases) of the Ethical Committee. The former was approved on 30 October 2012 and registered at ClinicalTrials.gov on 12 July 2018 with Identifier NCT03583723, whilst the latter was approved on 16 April 2019 with Identifier 16/19 OSS, and it was closed on April 2022. The Institutional Review Board approved this review. Written informed consent was obtained in all patients. The authors confirm that all ongoing and related trials for this intervention are registered.

The median OS for the entire population was 15.64 months, with a mean of 23.85 ± 77.22 (95% CI). The patients were then clinically followed until they were divided into two classes based on the median OS of all the patients: 95 dead and 96 alive.

### 2.1. Imaging

The characteristics investigated were extracted from CT scans collected at the time of patient diagnosis, on which expert radiation oncologists delineated the Clinical Target Volume (CTV).

For each patient, the CT images were acquired before the treatment using a Siemens Somatom Emotion, with 140 Kv, 80 mAs, and 3 mm for slice thickness. The scans were preprocessed applying a lung filter (kernel B70) and a mediastinum filter (kernel B31).

### 2.2. Clinical Features

Clinical data contained different information, which are listed in Table 2 together with the number of missing values and the distribution for each tabular feature among the different discrete values. To define the stage of the tumour, two experienced radiation oncologists (ROs) independently reviewed CT scans and assigned the staging scores of the tumour (T, N, and tumour stage); in case of disagreement, they reviewed the CT images together until consensus was reached. In addition to staging, age, and sex, Table 2 shows that we also collected features describing the histology of the tumour and the initial CTV, so that the clinical data account for seven descriptors in total.

In the imputation of missing values, the median value and the mode of the training set data were assigned for the numerical and categorical features, respectively. Furthermore, it should be noted that not all patients underwent a histopathological examination. Nevertheless, since on the one side it was not possible to impute the histology of the tumour and, on the other side, this feature could be informative, we add a virtual category named *unknown*.

## 3. Methods

**Figure 1:**
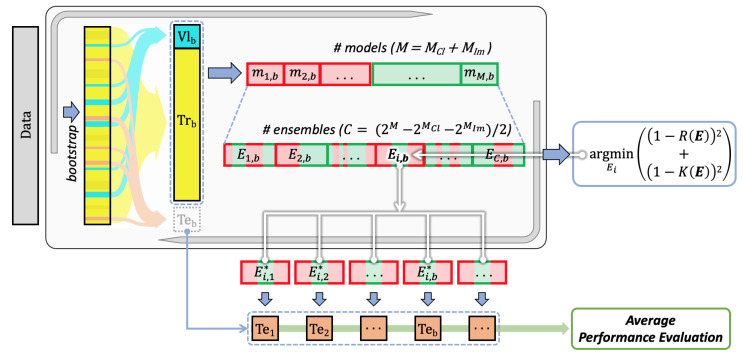
Schematic view of the pipeline. Symbols: Tr: training set, Vl: validation set, Te: test set, *m*: model, *M*: number of models ($\cdot_{Cl}$: for clinical data and $\cdot_{Im}$: for data from images), $\cdot_{b}$: a generic bootstrap fold, *E*: (models’) ensemble, $\cdot_{i}$: a generic ensemble, *C*: number of ensembles, *R*: function of recall, *K*: function of diversity.

To predict the prognosis in terms of binary classification task over the OS, we exploited both the images and clinical data described before, which were processed by a multimodal DL pipeline that, in the training phase, finds the optimal combination of models of different modalities via multiobjective optimisation. The idea stems from observing that today many deep neural networks are available, both in terms of architectures as well as of pretrained weights. This allows researchers to train or fine-tune them to search for the most suitable for the task at hand. Furthermore, it is well-known that, in many cases, ensembles of classifiers combined in late fusion provide better performance than unimodal models [33], but, at the same time, the learners in the ensemble have to complement each other, i.e., they have to make wrong decisions on different samples. Therefore, the abundance of available models asks for methods to support researchers in determining which is the best multimodal ensemble, a challenge that we address using an algorithmic and multimodal approach, schematically represented in Figure 1. It works with *m* different modalities and *M* different models, so that $M_{m}$ is the number of models available for the *m*th modality. Furthermore, we denote with *E* an ensemble built using one or more models per modality, whose outputs are combined by majority voting. Figure 1 shows that our method essentially consists of three main steps:

- Training all the available models for every single modality using the training sets defined by the bootstrap validation approach;
- Finding the multimodal set of unimodal models solving a multiobjective optimisation problem working with evaluation and diversity scores, which are computed on the validation sets defined by the same bootstrap approach;
- Computing the performance on the test sets defined by bootstrap, which are then averaged out (block “Average performance evaluation”).

These steps are now detailed in the next subsections.

### 3.1. Training

To obtain the optimal ensemble $E^{*}$ of models, the first step is to independently train and evaluate the different *M* unimodal models on the respective *m* modalities. In our scenario, we had $M=M_{Cl}+M_{Im}$, where $M_{Cl}$ and $M_{Im}$ denote the number of models for the clinical data and the imaging modality, respectively.

With respect to the clinical data, we worked with $M_{Cl}=7$ different ML and DL models, which are acknowledged in the literature as those that best work with this modality [34]. In alphabetical order they are:

- AdaBoost as a cascade of classifiers;
- Decision Tree (DT) as tree model;
- Multilayer perceptron (MLP) as neural architecture with one hidden layer with 13 neurons and 1 neuron in the output layer, which use the ReLU and Sigmoid activation functions, respectively;
- Random forest (RF) as an ensemble of trees;
- Support Vector Machine (SVM) as a kernel machine;
- TABNET [35] as a neural architecture;
- XGBoost a variation of the AdaBoost that uses a gradient descent procedure to minimise the loss when adding weak learners.

Let us now turn the attention to see image modality. We worked with $M_{Im}=30$ different CNNs from 8 architecture families, which have proved to have promising results in many biomedical applications [36]. They are:

- AlexNet [37];
- VGG [38]: VGG11, VGG11-BN, VGG13, VGG13-BN, VGG16, VGG16-BN, VGG19, VGG19-BN, where the suffix BN means that batch normalization is used;
- ResNet [39]: ResNet18, ResNet34, ResNet50, ResNet101, ResNet152, ResNeXt50, ResNeXt101, Wide-ResNet50-2, Wide-ResNet101-2;
- DenseNet [40]: DenseNet121, DenseNet169, DenseNet161, DenseNet201;
- GoogLeNet [41];
- ShuffleNet [42]: ShuffleNet-v2-x0-5, ShuffleNet-v2-x1-0, ShuffleNet-v2-x1-5, ShuffleNet-v2-x2-0;
- MobileNetV2 [43];
- MNasNet [44]: MNasNet0-5, MNasNet1-0.

All the CNNs were pretrained on the ImageNet dataset [45]. The architectures, layer organisation, and complexity of such models gave us the opportunity to investigate how different models perform on the task at hand.

### 3.2. Optimisation

To answer the question of which architectures should be used to construct the best multimodal ensemble, we solved a multiobjective optimisation problem that works with two scores capturing different views of the ensemble performance. Indeed, given an ensemble *E*, on one side we measured its recall (*R*) using, straightforwardly, the labels computed by applying the aforementioned majority voting scheme. *R* is defined as

(1) $$R=\frac{TP}{P}$$

where TP is the number of true positive classifications and *P* is the number of positive instances, and it measures the sensitivity of the model, a desirable property in our application ensuring that no positive patients get excluded before treatment. On the other side, the optimisation algorithm also works with the kappa diversity (*K*), a pairwise score measuring to what extent two models provide the same errors. It is defined as

(2) $$K=1-\frac{2(N_{11}N_{00}-N_{01}N_{10})}{\left(N_{11}+N_{10}\right)\left(N_{01}+N_{00}\right)+\left(N_{11}+N_{01}\right)\left(N_{10}+N_{00}\right)}$$

where $N_{11}$ and $N_{00}$ are the number of instances classified correctly and incorrectly by each of the two models under consideration, respectively, and $N_{10}$ and $N_{01}$ are the number of instances classified correctly by the first model and incorrectly by the second and vice versa, respectively. The overall ensemble diversity is given by

(3) $$\frac{2}{\left|E|(|E|-1\right)}\sum_{i=1}^{|E|-1}\sum_{j=i+1}^{\left|E\right|}k$$

where |E| is the number of models in *E*. Given these premises, let us notice that both *R* and *K* range in [0,1], and the higher the values, the more accurate and diverse the models. Hence, our algorithm solves the following multiobjective problem to determine the best ensemble $E^{*}$:

(4) $$E^{*}=\underset{E}{arg min}\left[{\left(1-R\left(E\right)\right)}^{2}+{\left(1-K\left(E\right)\right)}^{2}\right]$$

s.t.

(5) $$\left\{\begin{array}{c} |E^{*}|>1\\ |E^{*}|mod2=1\\ |E^{*}{|}_{Cl}\geq 1\\ |E^{*}{|}_{Im}\geq 1 \end{array}\right.\;$$

where R(E), K(E) represent the average values of *R* and *K*, respectively, of an ensemble *E* computed across all the validation sets given by bootstrap, a choice that avoids any bias. Looking at the constraints, $|E^{*}|$ denotes the number of models in $E^{*}$, whilst $|E^{*}{|}_{Cl}$ and $|E^{*}{|}_{Im}$ stand for the number of models in $E^{*}$ working with clinical and imaging data, respectively; finally, mod is the modulo operation. The first two conditions imply that the number of models in $E^{*}$ is odd to prevent ties in the majority voting, whilst the third and fourth conditions ensure that at least one model for each modality is present in $E^{*}$. Note also that finding $E^{*}$ is equivalent to finding the Pareto optimum of this optimisation problem, as we showed in [46,47,48]; nevertheless, here, we are extending our previous unimodal approach [46] to multimodal learning, as guaranteed by the last two conditions in Equation (5).

Hence, this optimisation algorithm performs an exhaustive search for the ensemble $E^{*}$ that, among the $C=\frac{2^{M}-2^{M_{Cl}}-2^{M_{Im}}}{2}$ combinations of learners, returns the best classification performance and reduces the incidence and effect of coincident errors among its members, thus considering possible relationships between models and modalities. Furthermore, the simple minimisation of only one of the objective functions (*R* or *K*) is not the best approach, since some models may degrade the performance of the ensemble, and they may have redundant classifications between each other, not exploiting the trade-off between performance and diversity [49].

Finally, in the test phase, each input instance is given to all the learners in $E^{*}$, whose outputs are combined by majority voting to obtain the final prediction.

### 3.3. Preprocessing

Before feeding the data to the models, a preprocessing phase was executed for both modalities.

With reference to the clinical data, we applied one hot encoding to categorical features, so that the original 7 features were mapped to 27 descriptors, which in practice were used as input to all the classifiers mentioned before for the clinical data. Furthermore, numerical features were normalised in [0,1]. No data augmentation was applied to the clinical data. For all clinical models listed in Section 3.1, the default parameters of the libraries were used.

With reference to the imaging modality, we used a U-Net to automatically align the images by detecting the region of interest of the scans by including the bounding cuboid segmenting the lungs. The U-Net architecture has proved to obtain good performance in many biomedical applications [50]. We trained this network on the TCIA publicly available dataset [27], which comprises 422 patients, and on a subset of our dataset, 125 patients whose lungs had already been delineated, with the goal of segmenting the lung pixels of each 2D slice. From this segmentation, we extracted the minimum bounding cuboid of the segmented volume, preventing any deformation once re-scaled. As input, the U-Net received 224 × 224 images, and it was trained with an Adam optimiser and with a Dice loss function. The batch size was set to 32, and the number of epochs was equal to 50, but an early stop criterion was triggered at 13th epoch. We assessed the performance of this network in holdout cross-validation, obtaining a Dice score and an intersection over union equal to 98.5 and 97.0, respectively, which we considered satisfactory for our task.

Let us now focus on the image classification stage. All the CNNs work with 2D images, i.e., at CT slice level, and they need an input of size 224x224. To this end, each slice of the segmented lungs was resized to 224x224 and normalised with a min–max scaler, bringing the pixel values between 0 and 1. Random data augmentation was applied to prevent overfitting of the CNNs: horizontal or vertical shift (−22≤ pixels ≤22), random zoom (0.9≤ factor ≤1.1), vertical flip, random rotation ($-15^{\circ }\leq$ angle $\leq 15^{\circ }$), and elastic transform (20≤α≤40, σ=7). The cross-entropy loss was used and was regulated by an Adam optimiser with an initial learning rate of 0.001, which is scheduled to reduce by an order of magnitude every time the minimum validation loss does not change for 10 consecutive epochs. For all the nets, a maximum of 300 epochs was fixed, with an early stopping of 25 epochs following the validation loss.

Given the fact that the CNNs work at slice level and the clinical data at patient level, to uniform the classifications, we aggregated the predictions of the slices of each patient via a majority-voting rule, thus obtaining a final outcome for each modality at patient level.

All the training processes were executed using an NVIDIA TESLA V100 GPU with 16 GB of memory, using PyTorch and Scikit-learn as the main coding library.

## 4. Results and Discussion

All the experiments were performed in bootstrap, performing five random extractions of the samples, where in each fold the proportions between the training, validation, and testing sets are 80%-10%-10%, respectively. Straightforwardly, in the imaging modality, all the slices coming from the same patient were always in the same set.

Table 3 shows the results: each row corresponds to a classifier in the case of unimodal learners reported in the uppermost section; it corresponds to a multimodal ensemble in the middle section, and it corresponds to a competitor in the bottom most section. The columns report the performance measured in terms of accuracy, F-score, and recall to have a complete view of how the different models perform on the test sets. With reference to the unimodal learners, the values in Table 3 show that the best classifier working with clinical data is the AdaBoost, whilst in the case of image data, the best CNN is the VGG11-BN. Both achieve the largest accuracy and F-score among the pool of unimodal models, whilst the latter is also the best in terms of recall.

The ensemble returned by our algorithm, denoted by $E^{*}$ in the table, achieves larger performance in terms of accuracy, F-score, and recall with respect to the unimodal classifiers. Whilst this could be expected in the case of the recall, as it is built maximising a function including this metric, it is interesting to note that this happens also in the case of the accuracy and F-score.

It is worth noting that the Pareto optimum $E^{*}$ is composed of three models (two from the imaging modality and one from the clinical modality): ResNet34, VGG11-BN, and TABNET, which belong to different families, suggesting that each model interprets its modality in a different way to address the classification task. We notice that $E^{*}$ has better performance (for all metrics) than the two best unimodal models. This finding implies that it is useful to fuse different modalities, each carrying useful and distinct information for the prognosis task whilst, at the same time, it is important to consider the diversity also, since it offers complementary points of view to the ensemble.

To assess the optimisation function, we also investigated which are the performances of the ensembles maximising only *R* or *K*, denoted as $E^{R}$ and $E^{K}$, respectively (middle section of Table 3). The former consists of DT, RF, and AlexNet, whereas the latter comprises DT, RF, and DenseNet161. Moreover, in this case, the class predictions on the test set revealed that the outputs provided by $E^{*}$ are better than those returned by $E^{R}$ and $E^{K}$. This finding supports the importance of satisfying the proposed multiobjective optimisation condition. This agrees with the literature that in other fields, and in the case of the majority voting rule, reports that a necessary and sufficient condition for an ensemble to be more accurate than any of its models is if the models are accurate and diverse [51].

To further prove the efficacy of the proposed approach, the middle section of Table 3 presents the performance of the following other experiments:

- $\bar{E}$: it denotes the average performance for all the possible ensembles;
- $E_{post}^{3}$: it denotes the performance of the ensemble consisting of the unimodal models with the largest recall, i.e., AdaBoost, ResNet34, and VGG11-BN. In this case, we adopt the subscript *post* to specify that such three models were *a posteriori* selected, i.e., they provide the largest performance on the test set, and not on the validation set;
- $\bar{E_{post}^{2+*}}$: it denotes the average performance attained by all the possible ensembles, including the two unimodal classifiers with the largest *a posteriori* recall, i.e., Adaboost and VGG11-BN, whilst varying the remaining experts included in the ensemble;
- $E^{ȼ}$: it denotes the performance of the ensemble obtained relaxing the multimodality constraints, and it is composed of AdaBoost, DT and RF.

It is worth noting that the performances returned by such four experiments are always lower than the performance of $E^{*}$ and even lower than several unimodal learners. This confirms, again, that maximising recall and diversity together is a useful driver to guide the ensemble set-up, i.e., to select which are the unimodal learners to be included. Furthermore, the fact that such ensembles in some cases provide lower performance than some unimodal learners confirms that handcrafted ensemble definitions can lead to sub-optimal results.

The last section of Table 3 presents a direct comparison of our approach with two state-of-the-art studies [23,24], which are denoted as DeepMMSA and MCF. As described in Section 1, they work with clinical and imaging modalities so that we can apply them to our data, computing the same scores we used for the other architectures under consideration. Note also that we do not experimentally evaluate [22,25] because, on the one side, ref. [22] works with CT and PET images and it is not designed to handle clinical data, whereas on the other side, ref. [25] does not work with CT images. The results show that such competitors perform worse than our method; we deem that this happens because such papers manually define the composition of the multimodal architectures, whilst our solution relies on an optimisation process.

As a further issue in our discussion, let us recall that three types of fusion exist in multimodal learning: early, joint, and late fusion. The latter is the one we used in this work, whilst the other two are other possible ways to proceed, which we considered as possible competitors for our method. To this end, we set up an early fusion learner using the best model per modality, i.e., AdaBoost and VGG11-BN, as already mentioned. Furthermore, we used the VGG11-BN as a feature extractor from the CT images, which we then concatenated with the clinical features and feed to the AdaBoost. We tested both slice-level and patient-level early fusion. The former consists of repeating the clinical features for each individual patient slice, whilst in the latter we averaged the CNN output of each slice to obtain a single feature vector per patient. These approaches got an accuracy equal to 62.92 ± 8.31% and 70.00 ± 9.35%, and an F-score equal to 62.69 ± 6.41% and 69.83 ± 12.25%, respectively, for the slice- and patient-level fusion, which are lower than the proposed approach. We did not perform any joint fusion since the best unimodal model (AdaBoost) has larger performance than a fully connected network (MLP). This, in turn, makes it not possible to apply joint fusion between the adaptive boosted ensembles and the VGG11-BN, although this is an issue worthy of investigation in a future work.

## 5. Conclusions

In this manuscript, we proposed a multimodal method for survival analysis of NSCLC. NSCLC has been already studied in a few other works employing multimodal learning but, differently from the literature, we propose an algorithm able to identify the optimal set of classifiers to be added to the multimodal ensemble in a late fusion approach. Our study is based on two modalities, clinical and CT imaging data, of a cohort of 191 patients suffering from locally advanced non-small-cell lung cancer.

From a clinical point of view, the possibility of having prognosis prediction tools in addition to clinical data, and especially before starting treatment, represents an unmet need of particular interest. If this data are available at the start of therapy, the treatment itself could be modified, adapting it to the expected response, thus intensifying or descaling therapy in patients with poor or good prognosis, respectively.

Indeed, we presented an optimised late fusion ensemble search method that finds the optimal combination of multimodal models considering both a metric of performance and a diversity score. Experimental results show that our method outperforms conventional unimodal models, bringing significant increase in performance in the multimodal ensemble. Among the different combinations of classification algorithms, the proposed approach achieves an accuracy of 75.00%, an F-score of 77.70%, and a recall of 84.00%, achieved using a ResNet34 and a VGG11-BN for the imaging modality and a TABNET for the clinical modality. A limitation of our approach is the need to train all models before the optimal set can be selected, which certainly represents a high computational cost.

The results described so far suggest four future directions worthy of investigation:

- Retrieving data at 1-, 2-, and 3-year time points as well as the progression free survival, which would add useful information;
- Provide more complementary information by adding other modalities to improve performance, such as WSI, genome sequencing, etc.;
- Perform different multimodality fusion approaches, such as joint fusion to obtain a end-to-end trainable system able to exploit the inherent correlations between multiple modalities;
- Search for an approach that a priori selects the models to be included in the ensemble, without the need to train them all individually;
- Switch from a classification to a regression task, which will allow predicting the actual survival time, also integrating the “Input doubling method” [52] as a preprocessing tool to augment the training set size.

## Figures and Tables

**Table 1:** Summary of the background on the multimodal learning to predict the overall survival in NSCLC. For the sake of completeness, the last section puts our contribution in the context of the literature.

Author	Modalities	Study Population	Number of Patients	Data Representation	Fusion Modality	Learning Model	Performance
Amini et al. [22]	CT, PET	NSCLC I-IV stages	182	Radiomic features extracted from an image obtained by merging PET and CT scans through a technique based on 3D discrete wavelet transform	Early	Kaplan–Meier method	C-index: 0.708
Wu et al. [23]	CT, clinical data	NSCLC I-III stages	422	Concatenation of deep features extracted by a 3D-ResNet34 and an MLP for CT images and clinical data, respectively	Early	MLP	C-index: 0.658
He et al. [24]	CT, clinical data	NSCLC I-III stages	316	Clinical data and radiomic features	Late	Modular architecture with SVM, DT, KNN, RF, and XGBoost as base classifiers	AUC: 0.81
Vale-Silva and Rohr [25]	clinical data, gene expression, microRNA expression, DNA methylation, gene copy number variation data, and WSI	33 different cancer types	11.081	Element-wise maxima across the set of representation vectors of single-modality submodels	Joint	Modular architecture, with dedicated input data modality submodels, a data fusion layer, and a final survival prediction MLP submodel	Time-dependent C-index: best 0.822 lung squamous cell carcinoma 0.554
*Putting our work in the background*	CT, clinical data	NSCLC II-IV stages	191	Clinical data and CT slices	Optimisation-driven late	multimodal ensemble of learners trained on different modalities and selected by a multiobjective optimisation algorithm	ACC: 0.75

**Table 2:** Patients’ characteristics. As marked by *, note that, although *age* and *CTV* are continuous variables, for the sake of synthesis we report here their distribution considering their median values as thresholds, whilst the model used the continuous values. The division into stages is further defined by letters (a, b, and c), which are not reported for the sake of brevity, but the model uses the actual stages.

Feature	Missing Data	Categories	Distribution
*Age* *	26 (13.62%)	<71 years	82 (42.93%)
		≥71 years	83 (43.46%)
*CTV* *	37 (19.37%)	<114.88 ${\mathrm{cm}}^{3}$	77 (40.31%)
		≥114.88 ${\mathrm{cm}}^{3}$	77 (40.31%)
*Sex*	0 (0.00%)	Male	133 (69.63%)
		Female	58 (30.37%)
*Histology*	0 (0.00%)	Adenocarcinoma	95 (49.74%)
		Squamous	59 (30.89%)
		Other	11 (5.76%)
		Unknown	26 (13.61%)
*Stage*	0 (0.00%)	II	4 (2.09%)
		III	160 (83.77%)
		IV	27 (14.14%)
*T stage*	36 (18.85%)	T0	1 (0.52%)
		T1	9 (4.71%)
		T2	32 (16.75%)
		T3	65 (34.03%)
		T4	48 (25.13%)
*N stage*	26 (13.61%)	N0	15 (7.85%)
		N1	33 (17.28%)
		N2	93 (48.69%)
		recurrence N2	6 (3.14%)
		N3	18 (9.42%)

**Table 3:** Performance of all the tested models with the best for each modality reported in bold. Each column shows the mean value of a performance metric followed by the standard deviation. $E^{*}$ is our optimum ensemble; $E^{R}$ and $E^{K}$ are the ensembles which maximise *R* and *K*, respectively; $\bar{E}$ is the average performance for all the possible ensembles; $E_{post}^{3}$ is the ensemble consisting of the unimodal models with the largest recall, $\bar{E_{post}^{2+*}}$ is the ensemble with the two unimodal classifiers with the largest recall per modality whilst varying the remaining experts included in the ensemble; $E^{ȼ}$ is the ensemble obtained relaxing the multimodality constraints.

Classifier	Modality	Accuracy	F-Score	Recall
**AdaBoost**	Clinical	**65.00 ± 5.00**	**67.35 ± 6.53**	**74.00 ± 16.73**
DT	Clinical	60.00 ± 3.54	59.42 ± 9.15	62.00 ± 20.49
MLP	Clinical	61.00 ± 5.48	54.37 ± 23.57	60.00 ± 38.08
RF	Clinical	60.00 ± 6.12	60.72 ± 9.74	64.00 ± 16.73
SVM	Clinical	59.00 ± 2.24	55.46 ± 10.29	54.00 ± 18.17
TABNET	Clinical	63.00 ± 10.37	64.68 ± 11.69	70.00 ± 22.36
XGBoost	Clinical	54.00 ± 8.22	49.67 ± 16.74	50.00 ± 24.49
AlexNet	Imaging	50.00 ± 0.00	0.00 ± 0.00	0.00 ± 0.00
DenseNet121	Imaging	62.00 ± 19.24	59.97 ± 27.79	66.00 ± 35.07
DenseNet161	Imaging	69.00 ± 6.52	68.28 ± 8.88	70.00 ± 20.00
DenseNet169	Imaging	71.00 ± 17.82	72.28 ± 17.44	76.00 ± 20.74
DenseNet201	Imaging	63.00 ± 16.05	65.95 ± 16.37	74.00 ± 23.02
GoogLeNet	Imaging	60.00 ± 6.12	50.04 ± 19.69	48.00 ± 31.14
MNasNet0-5	Imaging	51.00 ± 13.42	45.65 ± 19.37	44.00 ± 23.02
MNasNet1-0	Imaging	62.00 ± 7.58	65.11 ± 9.94	74.00 ± 20.74
MobileNetV2	Imaging	67.00 ± 17.18	68.61 ± 17.17	74.00 ± 23.02
ResNet101	Imaging	51.00 ± 5.48	49.97 ± 20.44	60.00 ± 38.08
ResNet152	Imaging	71.00 ± 7.42	63.65 ± 19.16	60.00 ± 30.82
ResNet18	Imaging	64.00 ± 18.84	58.74 ± 29.30	60.00 ± 33.91
ResNet34	Imaging	70.00 ± 11.73	71.71 ± 10.51	78.00 ± 22.80
ResNet50	Imaging	69.00 ± 11.40	69.45 ± 17.58	78.00 ± 27.75
ResNeXt101	Imaging	69.00 ± 7.42	68.95 ± 8.46	70.00 ± 15.81
ResNeXt50	Imaging	63.00 ± 10.37	64.35 ± 19.82	78.00 ± 33.47
ShuffleNet-v2-x0-5	Imaging	74.00 ± 10.25	74.66 ± 11.07	78.00 ± 16.43
ShuffleNet-v2-x1-0	Imaging	67.00 ± 17.18	67.14 ± 20.74	72.00 ± 26.83
ShuffleNet-v2-x1-5	Imaging	74.00 ± 13.87	72.3 ± 19.53	74.00 ± 27.02
ShuffleNet-v2-x2-0	Imaging	73.00 ± 9.08	71.23 ± 11.99	70.00 ± 20.00
VGG11	Imaging	50.00 ± 0.00	0.00 ± 0.00	0.00 ± 0.00
**VGG11-BN**	Imaging	**74.00 ± 16.36**	**75.03 ± 16.37**	**78.00 ± 19.24**
VGG13	Imaging	50.00 ± 0.00	0.00 ± 0.00	0.00 ± 0.00
VGG13-BN	Imaging	64.00 ± 8.22	61.58 ± 25.24	72.00 ± 35.64
VGG16	Imaging	50.00 ± 0.00	0.00 ± 0.00	0.00 ± 0.00
VGG16-BN	Imaging	71.00 ± 13.42	72.19 ± 10.95	74.00 ± 13.42
VGG19	Imaging	50.00 ± 0.00	0.00 ± 0.00	0.00 ± 0.00
VGG19-BN	Imaging	59.00 ± 15.17	51.68 ± 32.01	58.00 ± 38.99
Wide-ResNet101-2	Imaging	68.00 ± 10.95	69.84 ± 9.55	76.00 ± 20.74
Wide-ResNet50-2	Imaging	64.00 ± 13.87	66.02 ± 12.41	70.00 ± 18.71
***E*** ${}^{*}$	Multimodal	**75.00 ± 16.20**	**77.70 ± 13.83**	**84.00 ± 15.17**
$E^{R}$	Multimodal	60.00 ± 6.12	58.15 ± 9.40	58.00 ± 17.89
$E^{K}$	Multimodal	61.00 ± 5.48	62.02 ± 9.58	66.00 ± 16.73
$\bar{E}$	Multimodal	66.58 ± 11.30	61.44 ± 15.13	62.35 ± 22.00
$E_{post}^{3}$	Multimodal	72.00 ± 12.04	75.41 ± 10.68	83.00 ± 15.17
$\bar{E_{post}^{2+*}}$	Multimodal	70.94 ± 10.90	71.79 ± 10.21	74.91 ± 13.86
$E^{ȼ}$	Multimodal	61.00 ± 2.24	61.09 ± 8.11	64.00 ± 18.17
DeepMMSA [23]	Multimodal	59.00 ± 6.52	58.07 ± 12.32	52.00 ± 32.71
MCF [24]	Multimodal	62.00 ± 2.74	61.04 ± 10.53	64.00 ± 23.02

## Data Availability

The CLARO dataset is available upon request.

## Abbreviations

The following abbreviations are used in this manuscript:

AI	Artificial Intelligence
ML	Machine Learning
DL	Deep Learning
DT	Decision Tree
RF	Random Forest
MLP	Multilayer Perceptron
CNN	Convolutional Neural Network
SVM	Support Vector Machine
AUC	Area Under the ROC Curve
CT	Computed Tomography
MRI	Magnetic Resonance Imaging
PET	Positron Emission Tomography
WSI	Whole Slide Image
TCIA	The Cancer Imaging Archive
NSCLC	Non-Small-Cell Lung Cancer
OS	Overall Survival
CTV	Clinical Target Volume
